# The impact of price transparency of bundled vacation packages on travel decision making: An experimental study

**DOI:** 10.3389/fpsyg.2022.1053135

**Published:** 2022-12-23

**Authors:** Shizhen Bai, Lingyun Chu, Kim-Shyan Fam, Sheng Wei

**Affiliations:** ^1^School of Management, Harbin University of Commerce, Harbin, China; ^2^School of Foreign Languages, Harbin University of Commerce, Harbin, China

**Keywords:** price transparency, heuristic-systematic model, involvement, vertical position, purchase intention

## Abstract

Price transparency is a vital factor in consumers’ judgements and decisions. When selecting a bundled vacation package, travelers are often influenced by transparency in the prices of individual elements of the package. However, because of the diversity of elements bundled in a vacation package, it is a challenge to research the impact of price transparency. To try to overcome this challenge, our study used five experiments to examine the primary impact of element price transparency on travelers’ purchases, along with the moderating effects of consumer involvement and the vertical position of element prices in product descriptions. For the primary effect, we found that tourists preferred vacation packages with low transparency in element prices. We also found that the primary effect of price transparency remained consistent and robust across both revised and actual vacation packages. For moderating effects, we found that tourists with low involvement attached greater importance to price transparency than those with high involvement when the element price was presented higher in the product description of the travel package. The findings of the five experiments have theoretical implications for price transparency and Heuristic-systematic Model and practical implications for tourism professionals designing and marketing vacation packages.

## Introduction

In the past two decades, China’s tourism industry has progressed rapidly, and the number of tourists travelling with guided tour has also increased year by year.^1^ In 2018, 55.24% of the Chinese tourists purchased travel packages, and 50.65% of the Chinese respondents said they would like to participate in guided tours.^2^ With the control of epidemic prevention, China’s tourism industry has gradually recovered, and the number of orders for package tours in 2022 has increased significantly.^3^ This is determined by the fact that package tours allow tourists to travel at a relatively low price because of the bundling of air tickets, hotels, destinations and insurance (Footnote 1). Therefore, price plays a crucial part when tourists choose to purchase vacation packages. However, tourism enterprises seem to neglect the impact of price, which has jeopardized the reputation of some vacation packages in China, for example, the overcharging scandal in *China Snow Town* and the problem of different charges for the same package. The root cause of the above issues is closely related to the price transparency of vacation packages. The tourism enterprises attempt to benefit from information asymmetry by concealing the element prices in packages.

Tourism enterprises usually strategically manipulate (disclose or conceal) the element prices within a bundled vacation package. To make the best purchasing decision, tourists attempt to find relevant prices until they have formed some judgments (Hanna et al., 2019). They may look for element prices through various channels, such as websites or apps of airlines, hotels and destinations. However, this entails varying levels of efforts because of the diversity of elements in a travel package. In China, a vacation package is often bundled with elements such as transport (airplanes/trains), accommodation, destination visits and so on. Some prices are easy to find (e.g., tickets to resorts and fares), while others are not (rates of amenities and accommodation). In managing the trade-off between strategic manipulation of price and consumer decision-making, tourism managers and marketers must decide what they should reveal to effectively influence consumer purchase intentions. Serving as a sequence of examining the impacts of price transparency, this study sought to examine how price transparency of elements in a bundled vacation package is affecting travelers’ likeliness to purchase.

The existing research confirms the impact of price transparency on consumers’ positive responses. Miao and Mattila (2007) argue that price transparency can improve consumers’ perceived fairness. The research of Xia and Monroe (2004) confirms that price transparency can effectively stimulate consumers’ higher purchase intention by improving their price satisfaction. Tanford et al. (2012) demonstrates that the price transparency of the package has a positive impact on consumers’ perception of fairness, and thereby arouses consumers’ higher willingness to buy. However, few studies explore the impact of transparency in element prices in a package and reveal the mechanism and boundary conditions of the impact.

Above all, this paper explores the following questions through five experiments: (1) Under the circumstances of different levels of price transparency, how will consumers purchase travel packages? (2) How does consumer involvement affect the relationship between price transparency and consumer purchase intention, and will it play a moderating role? (3) How does the vertical position of an element price moderate the impact of price transparency on consumers’ purchase intention? Experiments 1, 2 and 3 mainly studied consumers’ willingness to purchase vacation packages under different levels of price transparency by changing experimental materials and participants. The main effects were verified through three experiments to ensure the external validity and robustness of the primary effect of price transparency, which lays the foundation for the subsequent tests of the moderating factors; Experiment 2 mainly examined how consumer involvement affected consumer responses to price transparency; Experiment 3 mainly investigated how the vertical position of an element price moderated the impact of price transparency on consumer decision-making. The conclusion of these experiments will provide some reference for travel agencies to make more effective price marketing strategies.

## Literature review

### Price transparency literature

Price transparency is an important dimension of information transparency and an extension of the core concepts of availability and accessibility (Zhu, 2002). Price transparency concerns how price information should be visible, based on the availability and accessibility of price information (Hanna et al., 2019). Granados et al. (2008) say that price transparency means consumers obtain price information (accessibility) about a seller’s products or services, which helps the buyer and seller determine the final transaction price (availability). Rossi and Chintagunta (2016) state that, considering the difficulty consumers have in obtaining price information, sellers usually selectively display the price of a specific product or service. On this basis, Hanna et al. (2019) proposed that price transparency is consumers’ perception of the difficulty of obtaining prices. Specifically, low transparency is the consumers’ perception that it takes a lot of time, energy and cognitive effort to obtain the price of a product and high transparency is their perception that it takes little time, energy and cognitive effort to obtain the price of a product.

As an important dimension of information transparency, existing research in the field of marketing mainly focuses on two issues: *whether* the price should be transparent and *how* the price should be made transparent. The first is mainly based on the accessibility and availability of price information, and the academic community has not reached a consensus on the impact. Some research shows that revealing the price of each component of the product would destroy the balance of information due to the spillover effect, and cause distortion to both parties of the transaction (Haviid and Mollgaard, 2006); Carlson and Weathers (2008) believe that price transparency has a negative impact on consumers’ perception of fairness and purchase intention. Other research indicates that price transparency can help enterprises:

- establish price advantages (Granados et al., 2008)
- generate welfare effects (Gu and Wenzel, 2011)
- increase consumers’ perception of fairness in higher prices (Miao and Mattila, 2007; Tanford et al., 2012)
- increase value perception (Xia and Monroe, 2004)
- improve consumer loyalty (Mittal and Agrawal, 2016; Peschel and Aschemann-Witzel, 2020)
- improve consumers’ attention on product performance (Chakravarti et al., 2002)
- stimulate consumers’ higher willingness to pay (Miao and Mattila, 2007), willingness to buy (Xia and Monroe, 2004), and willingness to pay a premium (Seim et al., 2017).

In tourism, it has been confirmed that price transparency benefits hotels by converting “lookers” to “bookers” of hotel rooms (Egger and Walters, 2008) and promoting the hotel revenue management (Noone, 2016). Additionally, Chin et al. (2022) posited that knowledge sharing (e.g., price transparency) may moderate the impact of tourism destination competitiveness on rural management sustainability. Price transparency may also profit tourism enterprises through precision marketing (Yamaura and Thompson, 2015) and relevance of response to negative online reviews, for example, negative reviews of price transparency (Kumar and Maidullah, 2022).

Regarding the question of how the price should be made transparent, Burman et al. (2016) suggested that, compared with high-priced hotels, low-priced hotels should adopt a strategy of high transparency for additional charges such as parking and telephone calls. In contrast to previous studies, Burman et al. (2016) focus on displaying the price of a certain type of products in the hotel, rather than the price of all products. However, this study does not clearly define “price transparency.” Based on the prior research, Hanna et al. (2019) redefined price transparency from the perspective of consumers’ perception of the difficulty of obtaining price information, and explained the impact of price transparency and price diversity on the choice of enterprise strategy. However, this research only discussed price transparency from a theoretical perspective, and did not carry out applied research. Zheng (2019) introduced the concept of price transparency to China and applied the concept to research on pricing strategy selection, but limited this to e-commerce platforms, and used the concept mainly for strategy interpretation without empirical research. Chu et al. (2022) conducted three experiments to examine the mechanism and boundary conditions of the impact of price transparency on consumers’ choice intention. However, this study was limited to online vacation packages, and to interpreting the influence mechanism of price transparency only from the perspective of marketing management.

Although previous research has shown that price transparency has an important impact on consumer behavior and decision-making, the academic community has not reached a unified research conclusion and relevant research on price transparency in the field of tourism remains insufficient. Our paper, therefore, takes travel packages as the research object, and takes price transparency as an important independent variable to investigate its influence and impact on consumer behavior and decision-making, with regards to willingness to purchase tourism products.

### The heuristic-systematic model of information processing

The heuristic-systematic model (HSM) is a theoretical model of the dual-processing of individuals when processing information (Chaiken, 1980; Eagly and Chaiken, 1993; Giner-sorolila and Chaiken, 1997). The model assumes that two information processing modes are usually adopted by individuals: systematic information processing and heuristic information processing. Systematic information processing describes that an individual, before or during decision-making, invests more time and cognitive effort in examining and testing persuasive information (Eagly and Chaiken, 1993). In contrast, heuristic information processing describes how individuals minimize cognitive effort to save time and energy by relying on heuristic cues in the context to make decisions (Chaiken et al., 1989). Although the heuristic information processing mode usually requires less effort and energy than systematic information processing, the heuristic system theory believes that the ability of individuals to conduct systematic information processing in real life is much lower than in laboratory experiments (Miao and Mattila, 2007). Therefore, as long as there are heuristic information cues, individuals will adopt the heuristic information processing mode (Chaiken et al., 1989).

HSM is a framework and behavioral paradigm for studying behavioral decision-making. It can explain individual processing, evaluating, using information and decision-making in different situations (He, 2021). It is widely used in the study of processing behaviors under different conditions and in the presence of different influencing factors (Chen, 2015). HSM has been applied in research into:

- disease risk (Trumbo, 1999; Trumbo, 2002; Etchegary and Perrier, 2007),
- computer and information technology (Winter, 2020; Zhang and Skoric, 2020),
- information security (Luo et al., 2013; Frauenstein and Flowerday, 2020),
- food safety (Kim and Paek, 2009; Chen, 2016),
- crisis management (Shi et al., 2020),
- decision-making (Allison et al., 1990)
- marketing field (Zhang et al., 2014; Lanero et al., 2020).

From research in the field of marketing, heuristic factors such as the reliability of information sources, and individual perception of information quality (Zhang et al., 2014) can stimulate purchase intention. In addition to research in the above fields, HSM is also used for the research in the fields of price and tourism. HSM is an effective method for describing decision-making based on price search (Darke et al., 1995). When an individual is in the systematic information processing mode, product information has a greater impact on individual judgment, while an individual’s judgment and evaluation in the heuristic information processing can be affected by price cues (Mitra, 1995), especially for low-involvement consumers who do not know much product information (Chung, 2013).

In tourism, HSM has served as the theoretical support for a number of studies, such as online reviews (Kim et al., 2017; Hlee et al., 2018; Ruiz-Mafe et al., 2018; Akhtar et al., 2020; Luo et al., 2021), online advertising (Bigne et al., 2021), AI recommendation (Li and Yang, 2017; Li et al., 2019; Shi et al., 2021), word of mouth (Bigne et al., 2020) and crisis communication in destinations (Cahyanto et al., 2016; Zhang et al., 2022). Extant research also centers on the heuristic cues. As results suggest, consumers’ heuristic processing can be activated through the sequence of positive and negative information (Ruiz-Mafe et al., 2018), time pressure (Bigne et al., 2021), rate of hotel rooms (Luo et al., 2021), social influence (Shi et al., 2020), website design (Kim et al., 2017), recommender identity disclosure (Li and Yang, 2017) and cost savings (Lee and Chung, 2019) and can further promote consumers’ likeliness to purchase.

The research above suggests that HSM provides a sound research perspective for the study of consumer decision-making. However, the impact of heuristic cues on sales performance, consumer decision-making and purchase intention has received relatively little attention from researchers (HLee et al., 2018). The impact of price transparency as a heuristic cue in consumers’ tourism product purchase intention is worthy of further discussion. Our paper, therefore, takes HSM as the theoretical basis to explore how varying levels of price transparency affect consumer decision-making around tourism products.

## Hypothesis development

### Price transparency and consumer purchase intention

We predicted that the transparency of element prices in a bundled vacation package might influence travelers’ purchase intentions. The framework of HSM strongly bolstered our prediction.

HSM argues that individuals are likely to process heuristic cues to save cognitive efforts in the decision-making process. This follows the principle of “minimum cognitive effort” (Chaiken et al., 1989). As a heuristic cue, price has been proved to influence judgement and evaluation of consumers (Mitra, 1995). Price transparency, as an attribute of price, is likely to activate heuristic processing of consumers and have impact on consumer responses. The diagnostic nature of heuristic cues can affect the process and the results of individual decision-making (Miao and Mattila, 2007). Cue diagnosticity and information diagnosticity refer to the extent to which consumers believe that cues or information are effective in purchasing decisions (Kempf and Smith, 1998). Regarding price transparency, consumers need to invest more cognitive effort in getting price information for products with low transparency. Therefore, consumers will perceive that the cues of low transparency prices have more reference value for decision-making and are more effective. Studies have shown that the higher the diagnostic ability of cues—that is, the higher the effectiveness of the cues—the more positive the response of consumers (Kim and Youn, 2019; Nedumkallel et al., 2020). In China, travel packages often bundle a variety of elements, and the difficulty of obtaining price information for each element is different—there are differences in the price transparency of different elements. In this situation, information with low transparency is more valuable than information with high transparency, and it is more likely to stimulate consumers’ purchase intention. Therefore, our hypothesis is:

*H1*: Bundled vacation packages with low transparency of prices will increase travelers’ purchase intention compared with those with high transparency prices.

### The moderating role of consumer involvement

Our research suggests that the factor of involvement affects the impact of price transparency. According to HSM, the choice of individual information processing mode (systematic, heuristic or both) depends on individual motivation and ability level. Only when individuals have strong motivation will they adopt systematic information processing (Todorov et al., 2002). Motivation is the desire to form an opinion consistent with the relevant facts, which will be enhanced when an individual personally involves (Trumbo, 2002). The lower the degree of individual involvement, the less likely it is to invest cognitive efforts in processing information (Chen and Chaiken, 1999). Therefore, individuals with low involvement tend to use heuristic cues.

Prior studies have shown that involvement can significantly affect people’s cognitive processes such as attention and memory, behavioral characteristics such as searching for product information and responses when processing information (Li and Liu, 2017). Research by Calvo-Porral et al. (2021) shows that low-involved consumers usually use heuristic information processing to process information related to product prices. According to this reasoning, involvement should be the core and key regulatory factor affecting purchase intention. Although heuristic processing and systematic processing can occur at the same time, when consumer involvement is low, consumers may choose to ignore the detailed description of travel packages and focus on the information of product prices as heuristic cues. Due to the principle of “minimum effort” (heuristic processing), heuristic cues may stimulate individuals’ heuristic information processing patterns. Individuals with high involvement, however, tend to carefully analyze and evaluate varied product information, while ignoring price-related information as heuristic cues (Chung, 2013).Therefore, our hypothesis states:

*H2*: Consumer involvement will moderate the impact of price transparency in a bundled vacation package on their purchase intention. Specifically, the positive effect of low transparency on purchase intention will be heightened when consumer involvement is low (vs. high).

### The moderating role of vertical position

Our study proposed that the impact of price transparency would be moderated by the vertical position of element prices in sales descriptions. Vertical position is a critical factor to arouse consumer’ attention in product selection both in conventional (Desmet and Renaudin, 1998) and in online retailing (Nordfalt et al., 2014; Septianto et al., 2020a, b). In the traditional retail setting, consumers pay more attention to the upper shelves, and top-shelf positions tend to elicit more positive brand evaluations (Chandon et al., 2009), higher quality perception(Machiels and Orth, 2017) and are more likely to be selected by consumers (Wongkitrungrueng et al., 2018). In online retail situations, consumers are more likely to select a certain product when it is displayed at the top of the computer screen, above all the other subsequent products (Breugelmans et al., 2007). In tourism, Ert and Fleischer (2016) demonstrate that the higher the hotel offering was presented in the list of hotel options, the more likely it was to be selected. Moreover, consumers consider products at the bottom in a display as less expensive and less trustworthy in quality (Valenzuela and Raghubir, 2015; Barbera et al., 2018). To summarize, a higher vertical position exerts more positive influence on consumer perception of a product and on the decision-making process than the lower vertical position. This may be partly explained by human reading habits. Psychology research posits that individuals have evolved to use a top-to-bottom reading sequence. The prediction, then, is that the prices positioned at the higher positions are more likely to attract consumer’ attention than those at the lower position and further enhance the impact of price transparency on consumer responses. Therefore, we propose the following hypothesis:

*H3*: The vertical position of prices in a vacation package will moderate the impact of price transparency on travelers’ purchase intentions. Specifically, the positive effect of low transparency prices on travelers’ willingness to purchase will be heightened when these prices are presented at the higher vertical position (vs. lower).

## Overview of empirical investigations

**Figure 1:**
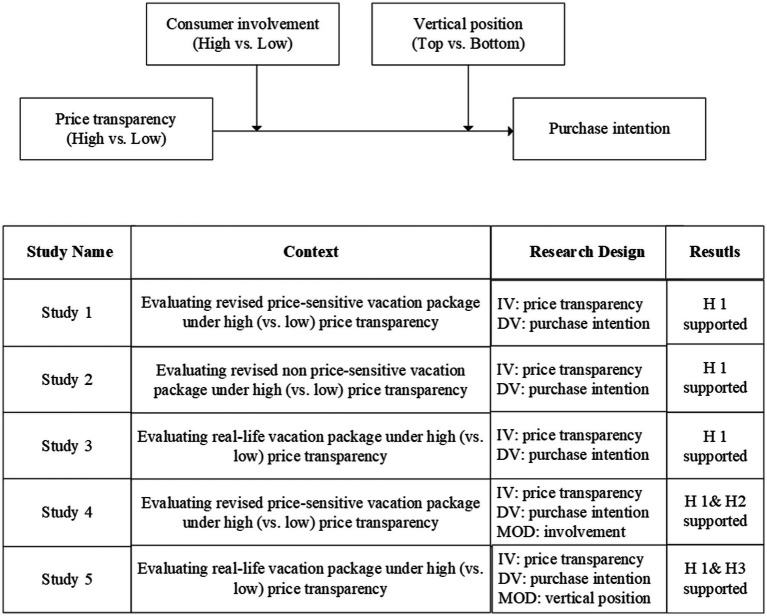
Theoretical framework and summary of empircal studies.

We conducted five experiments to empirically examine our hypotheses, and to guarantee internal and external validity and methods of measurement and manipulation. Three different experiment materials were used. The first, used on Experiments 1 and 4, was from the travel package *China Snow Town* in a local travel agency—chosen because it had been criticized for its price opaqueness. Although the destination had dealt with the price issue, the negative impact still exists and consumers are more sensitive to the package price. The product description and price information in the original material were revised. The second, used in Experiment 2, was from the vacation package of *Yalong Bay* as presented on a tourism website. This was chosen because it had a good reputation in terms of price and other aspects among tourists. Therefore, consumers were less sensitive to the price of this product. The original product description and price information were revised. The third material was *China Snow Town*, but in its original form rather than revised. This was chosen to ensure the external validity of the impact of price transparency. To minimize country-specific effects, only Chinese participants were recruited for the studies. Table 1 profiles the details of the participants. Figure 1 illustrates the conceptual model and gives a brief overview of the results of the experiments.

**Table 1:** Profiles of participants in studies 1–5.

		Study 1 (*N* = 163)	Study 2 (*N* = 306)	Study 3 (*N* = 298)	Study 4 (*N* = 222)	Study 5 (*N* = 282)
Gender	Male	52.3%	47.8%	50.9%	47.6%	52. 1%
	Female	47.7%	52.2%	49. 1%	52.4%	47.9%
Age	18–29	32. 1%	27.4%	18.0%	26.4%	23.6%
	30–39	30.6%	32.3%	35.4%	33. 1%	29.7%
	40–49	15.9%	18.7%	15.2%	19.0%	22.0%
	50–59	8.4%	11.5%	17.5%	12.0%	14.9%
	60-	13.0%	10. 1%	13.9%	9.5%	9.8%
Education level	Did not complete high school	1.5%	2.7%	2.3%	1.6%	0.5%
	High school graduate or some college	19.8%	22.3%	29.4%	35.2%	32.8%
	College graduate (4 years)	54.0%	58.9%	51.5%	49. 1%	51. 1%
	Postgraduate degree	24.7%	16. 1%	16.8%	14. 1%	15.6%
Personal expenditure per month	<3,000	14.3%	16.5%	12.7%	18.6%	19.0%
	3,001–5,000	44.2%	57.6%	56.9%	54.6%	61.2%
	5,001–8,000	13.2%	11.4%	16. 1%	8.9%	9.3%
	>8,001	16.7%	14.5%	14.3%	17.9%	10.5%

## Study 1: Examination of the main effect (h1)

Study 1 examined the main effect of the transparency of element prices in a vacation package on tourist purchase intention.

### Method: Subjects, and procedure

163 Chinese adult subjects (47.7% female, average age = 36.05) recruited from an online panel in March 2021, were randomly appointed to the two experimental conditions. (price transparency: high price transparency in Package A vs. low price transparency in Package B).

Subjects first imagined as requested that they were planning to take a packaged tour to *China Snow Town*. They were then required to read a revised vacation package description. We manipulated the presentation of different prices representing high and low transparency such that in one condition, Package A presented the tickets to resorts inside *China Snow Town*. In contrast, in another condition, Package B presented the prices of two amenities in it.

Then, subjects were demanded to indicate their purchase intention of the vacation package on three-item measurement rated using a five-point scale (i.e., 1 = “strongly disagree,” 5 = “strongly agree” ，Cronbach’s α = 0.819, Eisend, 2008). Finally, subjects were required to rate the perception of difficulty in seeking for the element prices presented in Packages A and B using a five-point scale (i.e., 1 = “very difficult,” 5 = “very easy”).

### Results and discussion

First, the manipulation of price transparency was successful, such that Package A was perceived as being highly transparent when it showed tickets to resorts (*M* = 3.54, *SD* = 0.989), compared with considering Package B less transparent when the package showed prices of amenities (*M* = 3. 16, *SD* = 0.935; *F* (1,161) = 6.377, *p* < 0.05, *η^2^* = 0.038).

Second, the participants’ purchase intention was significantly influenced by price transparency. Specifically, participants were more likely to purchase Package B with low transparency, where the prices of amenities were presented, (*M* = 3.925, *SD* = 0.758) than n Package A with high transparency, where the tickets to resorts were presented (*M* = 3.667, *SD* = 0.728; *F* (1, 161) = 4.902, *p* < 0.05, *η^2^* = 0.030).

With a between-subject experiment, study 1 provided initial support for H1, that is, low transparency will increase consumers’ purchase intention. To be specific, compared with the vacation package displaying element prices of high transparency (i.e., resort tickets in Package A), the vacation package displaying the element prices of low transparency(i.e., amenities charges in Package B) increased consumers’ likeliness to purchase the package.

## Study 2: Investigation of the mian effect (h1) in a different context

Study 2 set out to reproduce the result of the first experiment in the context of evaluating a coastal vacation package. The hypothesis of the main effect of price transparency was examined in a between-subjects design.

### Method: Subjects, and procedure

Subjects in the second study were 306 Chinse Adults (52.2%female, average age = 38.19) recruited from an online panel in July 2021.They were randomly appointed to one of the two experimental conditions (price transparency: high transparency in Package A vs. low transparency in Package B).

First, participants were provided information concerning how difficult they feel to discover the price of tickets to resorts and amenities by rating their perceived price transparency on a five-point scale (i.e., 1 = “very difficult,” 5 = “very easy”) in order to activate their perceived price transparency. Subjects were then asked to imagine that they were considering a package tour to a coastal city. The manipulation of price transparency was used the same way as that in the first study. Specifically in the high transparency scenario, Package A was described with the presentation of ticket to resorts, whereas in low transparency scenario, Package B was depicted with the presentation of prices of amenities. Subjects were then requested to answer questions to evaluate their likeliness to purchase.

### Results and discussion

First, we successfully manipulated price transparency in this study, such that Package A was perceived as being highly transparent, where tickets to resorts were displayed (*M* = 3.95, *SD* = 0.859), compared with considering Package B less transparent when the package showed prices of amenities (*M* = 3.66, *SD* = 1.008; *F* (1,304) = 7.274, *p* < 0.01, *η^2^* = 0.023).

Second, in the context of coastal vacation package, the impact of price transparency on consumers’ purchase intention was similar to that in study 1, that is, the participants’ purchase intention was significantly influenced by the price transparency manipulated in Packages A and B. To be specific, in Package B considered less transparent, where the prices of amenities were presented, consumers’ purchase intention was higher (*M* = 3.824, *SD* = 0.909) than Package A considered high transparent by displaying the tickets to resorts (*M* = 3.536, *SD* = 0.842; *F* (1, 304) = 8.204, *p* < 0 0.01, *η^2^* = 0.026).

In order to strengthen the external validity of the results, study 2 changed the experimental stimuli and the findings confirmed the positive impact of low transparency on intention to purchase vacation packages as predicted and H1 was further supported in a different context. Specifically, consumers were more likely to purchase Package B presenting the element prices of low transparency (i.e., amenities charges) than Package A presenting the element prices of high transparency (i.e., tickets).

## Study 3: Analysis of the main prediction (h1) with actual vacation packages

First, respondents were recruited from a local panel company to measure the perceived level of difficulty of acquiring prices in the given context. Then we concentrated on duplicating the results of the first two experiments to improve the generalizability of the findings. We also excluded the alternative explanation of consumers’ risk aversion.

### Method: Subjects, and procedure

Participants for manipulation check were 76 Chinese adults (48.0% female, average age = 38.56) recruited from a local panel company for a nominal payment in October 2021. Participants attended the study in a marketing laboratory room with computers. Respondents were asked to read the actual vacation package only with total price information of the vacation package listed as most of vacation packages do in China. Then respondents were asked to search for prices of ticket to resorts and amenities by using computers in order to activate their perceived price transparency. Finally, we measured their perceived level of difficulty of searching for price information by a five-point scale (i.e., 1 = “very difficult,” 5 = “very easy”).

The experiment recruited 298 Chinese adults (49. 1% female, average age = 40.21) from a local panel company. Subjects were appointed to two different experimental conditions (price transparency: high transparency in Package A vs. low transparency in Package B).First, we required the subjects to imagine that they were travelling to *China Snow Town*. After finishing the above tasks, participants were asked to evaluate their likeliness to buy the package by rating their purchase intention on a 5-point scale (i.e., 1 = “strongly disagree,” 5 = “strongly agree”) and to rate their risk aversion by responding to three 5-point items revised from Zhou et al. (2002) (Cronbach’s α = 0.724).

### Results and discussion

First, we successfully manipulated price transparency. Package A was perceived as high transparency where tickets to resorts were presented (*M* = 3.51, *SD* = 0.997) compared with Package B in which prices of amenities were displayed (*M* = 3.00, *SD* = 1.202; *F* (1, 74) = 4.117, *p* < 0.05, *η^2^* = 0.053).

Then, we conducted a main effect test by using one-way ANOVA with price transparency as the independent variable and consumers’ purchase intention dependent variable. The findings indicated that price transparency negatively influenced consumers’ purchase intention. Specifically, low transparency activated higher purchase likeliness (*M* = 3.737, *SD* = 0.921) than high transparency (*M* = 3.473, *SD* = 0.961, *F* (1, 296) = 5.847, *p* = 0.016, *η^2^* = 0.20).

As far as the alternative explanation of risk aversion is concerned ，the findings indicated that price transparency did not have impact on risk aversion (*M_high-transparency_* = 4.987, *M_low-transparency_* = 4.964, *t* (220) = 0. 162, *p* = 0.872). In addition, we find that there was no significant correlation between risk aversion and consumers’ purchase intention either at the overall level (*r* = −0.126， *p* = 0.069) or in high-transparency group (*r* = −0.094, *p* = 0.340) as well as low-transparency group (*r* = −0.168, *p* = 0.089).

Unlike studies 1 and 2 where revised descriptions of vacation packages were used, study 3 adopted the actual vacation package without any revision to testify the primary effect of price transparency in a real-life context. The findings provided support for H1, that is, the positive impact of low transparency on consumer’s purchase intention and the result remained consistent throughout the three studies. Additionally, study 3 found that there was no link between risk aversion and price transparency and purchase intention. Thus the alternative explanation of risk aversion was excluded.

## Study 4: Examination of the moderating effect of involvement

Study 4 followed the procedure of study 1 and used the same experimental stimuli as study 1. Additionally, the moderating effect of travelers’ involvement was investigated. It was predicted that the impact of price transparency on consumer purchase intention would be valid only for low-involved tourists.

### Method: Subjects, and procedure

222 Chinese adult subjects in the experiment were (52.4%female, average age = 38.98) recruited from an online panel. Generally, the procedure of study 4 replicated study 1 but made some alternations. Subjects were asked to seek for the price information of tickets to resorts and amenities in order to activate their different perceptions of price transparency. After that, they were told to imagine that they were planning to travel to *China Snow Town*. They were presented with two vacation packages: Package A with tickets to resorts listed and Package B with prices of amenities listed. Then as requested, subjects rated their purchase intention along a 5-point scale (i.e., 1 = “strongly disagree,” 5 = “strongly agree”).

Finally, subjects were required to rate their involvement in four items revised from the study of Habib et al. (2021) (e.g., I am happy about the advertisement of the product and likely to be more fond of it; I tend to make careful comparison between different products for its quality before I buy it; I attempt to seek for the product information in various ways before I decide to buy the product; I am greatly involved in the advertisement and not willing to miss the chance in making up mind to buy the vacation package.) using a 5-point scale(1 = “strong disagree,” 5 = “strongly agree,” Cronbach’s α = 0.687) and rate their perception of difficulty in acquiring prices of tickets and amenities.

### Results and discussion

After measurement, Package A displaying tickets to resorts was higher in price transparency (*M* = 3.94, *SD* = 0.877) than Package B displaying price of amenities in the vacation package (*M* = 3.02, *SD* = 1.236, *t* (220) = 6.390, *p* < 0.001). It provided evidence for the successful manipulation of price transparency.

A moderation test was implemented using Hayes (2017) process analysis with Model #1(i.e., independent variable: price transparency; moderator: involvement; dependent variable: purchase intention). The results demonstrated that the moderating role was negatively significant (effect = −0.986, *t* = −2.603, *p* = 0.010, 95% confidence Level [CI]: [−0. 173, −0.024]). Specifically, when participants’ involvement was relatively high (+1SD measurement), we found no significant impact of price transparency on consumers’ purchase intention (effect = 0.021, *t* = 0.516, *p* = 0.607, 95%CI [−0.059, 0.010]). In contrast, when participants’ product involvement was relatively low (− 1SD measurement), there was a significant impact of price transparency on consumers’ likeliness to purchase the package (effect = 0.218, *t* = 3.320, *p* = 0.001, 95%CI [0.089, 0.347]). The result confirms the existing conclusion that the evaluation of an advertised information are significantly higher for low-involved consumers (Chandrashekaran, 2004).

To investigate the moderating effect of consumer involvement, study 4 conducted a between-subject experiment and the result supported the moderating effect of consumers’ involvement on the influence of price transparency of vacation packages. Specifically, the impact of low transparency on consumer purchase intention were significant only when the consumer involvement was low. There was no significant difference on the impact of price transparency on purchase intention when consumer involvement was high.

## Study 5: Demonstration of the moderating evidence of vertical position (h3)

To examine the moderating effect of the vertical positions of prices, study 5 followed the procedure of study 3 with some modifications. Specifically, we expected that the findings of studies 1, 2, & 3 would be effective only when the element prices were positioned at the higher place, and less or no impact would be expected when the prices were placed at the lower position.

### Method: Subjects, and procedure

We recruited 282 Chinese adults subjects (47.9% female, average age = 41.63) from an online panel. Though the procedure of study 5 shares similarities with that of study 3, there were the following alterations. In this study we utilized a 2 (price transparency: high vs. low) × 2 (vertical position: top vs. bottom) between-subject experimental design. We presented participants with an actual vacation package of *China Snow Town*.

We manipulated price transparency in the same manner as Study 3. Vertical position was manipulated in the way that the prices of tickets and amenities were placed at the top and at the bottom in the product description. After reading the experimental stimuli, subjects were requested to evaluate their willingness to buy the vacation package using a 5-point scale (1 = “strongly disagree,” 5 = “strongly agree”).

### Results and discussion

We successfully manipulated price transparency. Package A which presents the tickets to resorts was perceived as being highly transparent (M = 3.78，SD = 0.952) compared to Package B which presents prices of amenities in the vacation package perceived as less transparent (M = 3.48 ，SD = 1.116, t (280) = 2.415, *p* = 0.016).

**Figure 2:**
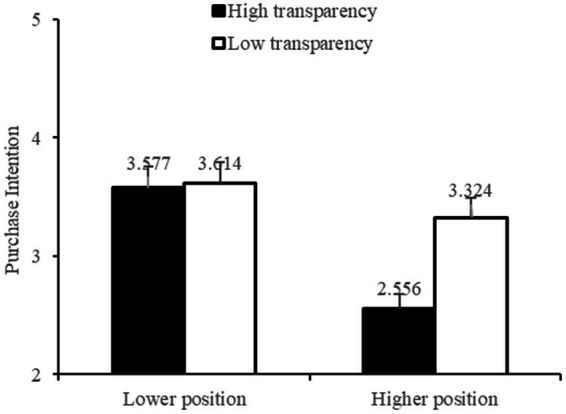
Results of study 5.

A moderation test was implemented using Hayes (2017) process analysis with Model #1(i.e., independent variable: Price transparency, moderator: vertical position; dependent variable: purchase intention). The results demonstrated that price transparency negatively influenced consumer purchase intention (effect = −0.768, *t* = −4.690, *p* = 0.00, 95% confidence level: [−1.10, −0.450]). H1 was supported. Moreover, the overall moderation was significant (effect = 0.731, *t* = 3.345, *p* = 0.001, 95% CI: [0.301, 1.162]). To be specific, When the price was positioned vertically higher, the purchase intention was higher when the price transparency was lower (*M* = 3.324, *SD* = 0.942) versus higher (*M* = 2.556, *SD* = 0.883, *p* = 0.00).When the price was positioned vertically lower, it was demonstrated that there was no significant impact of price transparency on purchase intention (*M_high-transparency_* = 3.577, SD = 1.064, *M_low-transparency_* = 3.614, *SD* = 0.703, *p* = 0.780) as shown in Figure 2.

In study 5, the researchers adopted a two-way between-subject experimental design by manipulating the variables of price transparency and vertical position to investigate the moderating effect of vertical position on the impact of price transparency on purchase intention. The results of study 5 supported H3 and demonstrated that vertical position of prices moderated the impact of price transparency on consumer intention to purchase vacation packages. Specifically, when the price of amenities (low transparency) was displayed higher in the package description, the impact of price transparency on consumer purchase intention was significant. When the price of amenities (low transparency) was displayed lower in the package description, there was no significant difference in the impact of price transparency on consumers’ willingness to buy. Therefore, the vertical position of prices moderates the impact of price transparency and H3 was supported.

## Conclusion and contributions

### Conclusion

This paper explored how the price transparency of a bundled vacation package influences travelers’ decision-making. We predicted that tourists would be less likely to buy a package when price transparency was high. Experiment 1 examined the main effect of the impact of price transparency (high vs. low) on purchase intention. Participants imagined travelling to *China Snow Town*, choosing between vacation packages of high and low transparency. The result of the second experiment, conducted for a coastal destination, was consistent with that of Experiment 1. Given the generalizability of the primary prediction, Experiment 3 found that the main effect of price transparency on consumer judgement was consistent and robust across revised and actual vacation packages, and the study excluded the alternative explanation of consumers’ risk aversion. Experiment 4 replicated Experiment 1 for packages to *China Snow Town* but with different price transparency. This supported the moderating effect of involvement on the previous findings. Experiment 5 replicated Experiment 3, showing the moderating role that vertical position played. Consumer likeliness to purchase a package with low-transparency price information increased significantly for participants whose involvement was low and when the price was presented vertically higher.

### Theoretical contributions

These results make contributions to the development of related theories. The study contributes to the stream of literature on price transparency. Zhu (2002) conceptualized price transparency as the availability and accessibility of price-relevant information, based on which studies were conducted to address the problem whether the prices of components or elements in a product or a bundle should be revealed. Hanna et al. (2019) redefined the construct of price transparency as the consumers’ perception of difficulty in seeking for prices of components in a product or prices of items in a bundle and moreover two types of price transparency were classified according to the amount of efforts that consumer invest in seeking for prices. Based on this, our research was to investigate the effect of price transparency on consumer decision-making, which contribute to the stream of literature on price transparency in the following aspects. The study extends our understanding of price transparency. It asserts that price transparency proposed by Hanna et al. (2019) is not only an attribute in a product but also a variable that can be manipulated. Moreover, the study confirms the ways to manipulate the variable of price transparency in the context of vacation packages. Our research, therefore, sheds new light on price transparency and sets the scene for future research.

Our findings also contribute to the literature on the heuristic-systematic model (HSM). In past literature, HSM has often been used in information system and marketing research to explain how individuals process information (Chaiken et al., 1989; Miao and Mattila, 2007). In our research, we found that travelers tended to select vacation packages where there was low transparency of element price. This is because travelers tend to invest more mental effort and time in finding a fair price where transparency is low rather than high. We therefore identified a crucial factor, whereby transparency offers more value as a heuristic cue to influence individual judgment. Moreover, the findings also contribute to the application of the heuristic-systematic model (HSM) to different contexts. It has been argued that traveler behavior has been influenced significantly because of the Covid-19 prevention and control polices (Zheng et al., 2021). Chen and Li (2022) suggests that future research should adopt experimental methods to study and find the appropriate psychological theories to explain the changes in traveler behavior under the influence of the pandemic. The research, exploring the causal link between price transparency and consumer response with experiments, responds to the academic demand and expands the application of Heuristic-systematic Model. Given the influence of Covid-19 pandemic, our future work will further explore the link between price transparency and consumer responses through experiments and reinforce the explanatory power of HSM in marketing research.

Our research also extends the literature concerning the impact of price transparency on behavioral pricing. It has long been debated whether price transparency is a disadvantage or an advantage for firms. On the negative side, previous research has argued that price transparency can harm an organization because it weakens customer loyalty and stimulates perceptions of price unfairness (Sinha, 2000; Zhu, 2002, 2004). Kuah and Weerakkody (2015) concluded that it was hard to define and identify price information in practice, and therefore price transparency could mislead consumers. In contrast, other literature has shown that a product presented with all its component prices could be more attractive to consumers and raise their intention to buy (Mohan et al., 2020). Simintiras et al. (2015) argued that price transparency helped consumers perceive fairness of price and greatly assisted them in making judgements about product quality.

By extending these investigations to the decision-making process under different price transparency situations, we found that individual purchase intention increased in the case of low transparency of element prices in a bundled vacation package. We thoroughly investigated these impacts through the *China Snow Town* package, a package to a coastal destination and an actual vacation package presented by a local travel agency. Our findings from the experiments also revealed that the construct of price transparency could be manipulated by presenting ticket prices for scenic destination (high price transparency) and the cost of amenities (low price transparency) in a vacation package.

### Practical contributions

This research offers practical and managerial implications. First, most studies have reached the consensus that transparency of element prices in packages can have a critical and positive influence on consumers’ evaluations (Morwitz et al., 1998; Chakravarti et al., 2002; Arora, 2011). Our study found that travelers tended to purchase bundled vacation packages that presented element prices that were hard or impossible to discover, process and compare. According to the results of multiple experiments for this paper, transparent amenity prices in a package were effective in persuading consumers. Therefore, one strategy for tourism marketers would be to display price of amenities in the package to increase likeliness to purchase.

Second, online and offline travel agencies need to be aware that the position of price information in descriptions of bundled vacation packages can play an important role in changing consumer behavior. Our research found that when the element prices were positioned at the higher place, the impact of price transparency was significant and thus suggests marketers should place the price information of low transparency higher in the marketing information to enhance purchase intentions.

Third, presenting the element prices of low transparency in a vacation package would be useful for potential consumers with little or no knowledge or prior experience of the package. Experiment 4 demonstrated that travelers’ involvement significantly changed the impact of price transparency on their choices. Specifically, travelers’ preference for packages with low transparency of prices was reinforced when their involvements were low. Our research suggests that tourism managers and marketers should attract low-involved travelers by taking advantage of prices of low transparency.

Fourth, the Covid-19 Pandemic has dealt a heavy blow to China’s tourism industry (Li et al., 2022). The domestic demand for travel was suppressed for two main reasons: first, the normalized control of the epidemic caused many inconvenience to travel and additionally the overall domestic economic situation has declined, and personal income has also decreased, which has affected the travel demand of consumers. In order to recover and improve business performance as soon as possible, tourism enterprises use various marketing methods to stimulate consumers to respond positively to vacation packages (Liu et al., 2022). In this context, this study has developed a reasonable price strategy for tourism enterprises through different price combinations, which can effectively stimulate tourists’ demand, improve tourists’ purchase intention, and promote the steady development of the tourism industry.

## Limitations and suggestions for future studies

There are some limitations in this study. First, Even if the study explores the moderating roles of involvement, there may be other cognitive factors,which were found to influence the impact of price on consumer responses, to be considered in our future work, such as travel goals (Kim et al., 2020), sense of power (Yao et al., 2020) and rationalism (Kim et al., 2022). Second, our findings only provided support for *vertical* position on the impact of price transparency. Since the *horizontal* placement affects consumer’s attitude (Chae and Hoegg, 2013), it would, therefore, be noteworthy to investigate whether the horizontal position of prices may influence the impact of price transparency on consumer’s decision-making. Finally, the participants were recruited online due to the Covid-19 prevention and control policies, which might affect the application of the results. Therefore future studies may conduct offline experiments to modify the model.

## Data availability statement

The original contributions presented in the study are included in the article/Supplementary material, further inquiries can be directed to the corresponding author.

## Author contributions

All author contributed to the background, conception and design of the study. LC performed the research process analysis and wrote the first draft of the manuscript. All author contributed to the manuscript revision, read, and approved the submitted version.

## Funding

The authors acknowledge the support from National Social Science Fund of China (grant number: 22BJY157), Social Science Found of Heilongjiang Province (grant number: 20GLH028), 2021 Harbin University of Commerce Teaching Staff “Innovation” Support Project (grant number: 20GLH028) and Philosophy and Social Sciences project of Heilongjiang Province (20GLE389).

## Conflict of interest

The authors declare that the research was conducted in the absence of any commercial or financial relationships that could be construed as a potential conflict of interest.

## Publisher’s note

All claims expressed in this article are solely those of the authors and do not necessarily represent those of their affiliated organizations, or those of the publisher, the editors and the reviewers. Any product that may be evaluated in this article, or claim that may be made by its manufacturer, is not guaranteed or endorsed by the publisher.

## Footnotes

^1^Insight And Info. (2018). Analysis of Domestic Guided Tour. https://market.chinabaogao.com/gonggongfuwu/031332440h018.html [Accessed March 13, 2018].

^2^Beijing News. (2019). Launching of Big data of Guided Tour. https://baijiahao.baidu.com/s?id=1640652573489514760&wfr=spider&for=pc [Accessed August 1, 2019].

^3^Tuniu Broadcasting. (2022). Report of Summer Tours in 2022. https://view.inews.qq.com/a/20220831A07SQ600 [Accessed August 31, 2022].
